# Immune digital twins for complex human pathologies: applications, limitations, and challenges

**DOI:** 10.1038/s41540-024-00450-5

**Published:** 2024-11-30

**Authors:** Anna Niarakis, Reinhard Laubenbacher, Gary An, Yaron Ilan, Jasmin Fisher, Åsmund Flobak, Kristin Reiche, María Rodríguez Martínez, Liesbet Geris, Luiz Ladeira, Lorenzo Veschini, Michael L. Blinov, Francesco Messina, Luis L. Fonseca, Sandra Ferreira, Arnau Montagud, Vincent Noël, Malvina Marku, Eirini Tsirvouli, Marcella M. Torres, Leonard A. Harris, T. J. Sego, Chase Cockrell, Amanda E. Shick, Hasan Balci, Albin Salazar, Kinza Rian, Ahmed Abdelmonem Hemedan, Marina Esteban-Medina, Bernard Staumont, Esteban Hernandez-Vargas, Shiny Martis B, Alejandro Madrid-Valiente, Panagiotis Karampelesis, Luis Sordo Vieira, Pradyumna Harlapur, Alexander Kulesza, Niloofar Nikaein, Winston Garira, Rahuman S. Malik Sheriff, Juilee Thakar, Van Du T. Tran, Jose Carbonell-Caballero, Soroush Safaei, Alfonso Valencia, Andrei Zinovyev, James A. Glazier

**Affiliations:** 1 Molecular, Cellular and Developmental Biology Unit (MCD), Centre de Biologie Integrative (CBI), University of Toulouse, UPS, CNRS, Toulouse, France; 2 Lifeware Group, Inria, Saclay-île de France, Palaiseau, France; 3 Department of Medicine, University of Florida, Gainesville, FL USA; 4 Department of Surgery, University of Vermont Larner College of Medicine, Vermont, USA; 5 Faculty of Medicine Hebrew University, Hadassah Medical Center, Jerusalem, Israel; 6 UCL Cancer Institute, University College London, Paul O’Gorman Building, 72 Huntley Street, London, WC1E 6BT UK; 7 Department of Clinical and Molecular Medicine, Norwegian University of Science and Technology, Trondheim, Norway; 8 The Cancer Clinic, St Olav’s University Hospital, Trondheim, Norway; 9 Department of Biotechnology and Nanomedicine, SINTEF Industry, Trondheim, Norway; 10 Department of Diagnostics, Fraunhofer Institute for Cell Therapy and Immunology, Leipzig, Germany; 11 Institute of Clinical Immunology, Medical Faculty, University Hospital, University of Leipzig, Leipzig, Germany; 12 Center for Scalable Data Analytics and Artificial Intelligence (ScaDS.AI), Dresden/Leipzig, Germany; 13 Department of Biomedical Informatics & Data Science, Yale School of Medicine, New Haven, CT USA; 14 Prometheus Division of Skeletal Tissue Engineering, KU Leuven, Leuven, Belgium; 15 Skeletal Biology and Engineering Research Center, Department of Development and Regeneration, KU Leuven, Leuven, Belgium; 16 Biomechanics Research Unit, GIGA Molecular and Computational Biology, University of Liège, Liège, Belgium; 17 Faculty of Dentistry Oral & Craniofacial Sciences, King’s College London, London, UK; 18 Biocomplexity Institute and Department of Intelligent Systems Engineering, Indiana University, Bloomington, Indiana, 47408 USA; 19 Center for Cell Analysis and Modeling, UConn Health, Farmington, CT 06030 USA; 20 Department of Epidemiology, Preclinical Research and Advanced Diagnostic, National Institute for Infectious Diseases ‘Lazzaro Spallanzani’ - I.R.C.C.S., Rome, Italy; 21 Mathematics Department and Center of Mathematics, University of Beira Interior, Covilhã, Portugal; 22 Barcelona Supercomputing Center (BSC), Barcelone, Spain; 23 Institute for Integrative Systems Biology (I2SysBio), CSIC-UV, Valencia, Spain; 24 Institut Curie, Université PSL, F-75005 Paris, France; 25 INSERM, U900, F-75005 Paris, France; 26 Mines ParisTech, Université PSL, F-75005 Paris, France; 27 Université de Toulouse, Inserm, CNRS, Université Toulouse III-Paul Sabatier, Centre de Recherches en Cancérologie de Toulouse, Toulouse, France; 28 Department of Biology, Norwegian University of Science and Technology, Trondheim, Norway; 29 Department of Mathematics and Statistics, University of Richmond, Richmond, VA USA; 30 Department of Biomedical Engineering, University of Arkansas, Fayetteville, AR USA; 31 Interdisciplinary Graduate Program in Cell and Molecular Biology, University of Arkansas, Fayetteville, AR USA; 32 Cancer Biology Program, Winthrop P. Rockefeller Cancer Institute, University of Arkansas for Medical Sciences, Little Rock, AR USA; 33 Department of Mechanical and Aerospace Engineering, University of Florida, Gainesville, FL USA; 34 Maastricht Centre for Systems Biology (MaCSBio), Maastricht University, Maastricht, The Netherlands; 35 INRIA Paris/CNRS/École Normale Supérieure/PSL Research University, Paris, France; 36 Andalusian Platform for Computational Medicine, Andalusian Public Foundation Progress and Health-FPS, Seville, Spain; 37 Bioinformatics Core Unit, Luxembourg Centre of Systems Biomedicine LCSB, Luxembourg University, Esch-sur-Alzette, Luxembourg; 38 Department of Mathematics and Statistical Science, University of Idaho, Moscow, ID 83844-1103 USA; 39 Novadiscovery, Lyon, France; 40 Department of Electrical and Computer Engineering, University of Patras, Patras, Greece; 41 Department of Bioengineering, Indian Institute of Science, Bengaluru, India; 42 School of Medical Sciences, Faculty of Medicine and Health, Örebro University, SE-70182 Örebro, Sweden; 43 X‐HiDE - Exploring Inflammation in Health and Disease Consortium, Örebro University, Örebro, Sweden; 44 Multiscale Mathematical Modelling of Living Systems program (M3-LSP), Kimberley, South Africa; 45 Department of Mathematical Sciences, Sol Plaatje University, Kimberley, South Africa; 46 Private Bag X5008, Kimberley, 8300 South Africa; 47 European Bioinformatics Institute, European Molecular Biology Laboratory (EMBL-EBI), Hinxton, Cambridge, UK; 48 Department of Surgery and Cancer, Faculty of Medicine, Imperial College London, London, UK; 49 Department of Microbiology & Immunology and Department of Biostatistics & Computational Biology, University of Rochester Medical Center, Rochester, NY 14642 USA; 50 Vital-IT Group, SIB Swiss Institute of Bioinformatics, Lausanne, Switzerland; 51 Institute of Biomedical Engineering and Technology, Ghent University, Gent, Belgium; 52 Auckland Bioengineering Institute, University of Auckland, Auckland, New Zealand; 53 ICREA, 23 Passeig Lluís Companys, 08010 Barcelona, Spain; 54 In silico R&D, Evotec, 31400 Toulouse, France

**Keywords:** Computational biology and bioinformatics, Computer modelling

## Abstract

Digital twins represent a key technology for precision health. Medical digital twins consist of computational models that represent the health state of individual patients over time, enabling optimal therapeutics and forecasting patient prognosis. Many health conditions involve the immune system, so it is crucial to include its key features when designing medical digital twins. The immune response is complex and varies across diseases and patients, and its modelling requires the collective expertise of the clinical, immunology, and computational modelling communities. This review outlines the initial progress on immune digital twins and the various initiatives to facilitate communication between interdisciplinary communities. We also outline the crucial aspects of an immune digital twin design and the prerequisites for its implementation in the clinic. We propose some initial use cases that could serve as “*proof of concept*” regarding the utility of immune digital technology, focusing on diseases with a very different immune response across spatial and temporal scales (minutes, days, months, years). Lastly, we discuss the use of digital twins in drug discovery and point out emerging challenges that the scientific community needs to collectively overcome to make immune digital twins a reality.

## Building a sustainable interdisciplinary community of researchers focused on Immune Digital Twin (IDT) technology

A digital twin (DT) in biomedicine is a virtual representation of a patient, or a patient’s state, that allows communication and data feedback from the actual patient to the virtual patient and vice versa. This capability holds the potential to improve personalised care and patient-tailored treatments. However, implementing such a technology may only be feasible for some pathologies. A recent report by the National Academies of Sciences, Engineering, and Medicine (NASEM) in the US specified that in healthcare, this feedback loop might not be through (semi-)automated interactions but might require a human-in-the-middle^1^. This interpretation aligns with the definition taken by the European Commission in developing their Virtual Human Twin (VHT) initiative and the recommendations in the VHT roadmap^2,3^. While DT approaches in medicine are still in their infancy, a few biomedical applications close to the DT concept have already been implemented in oncology, radiology, and cardiology^4–18^. DTs of large blood vessels could allow the early diagnosis of potential abnormalities and aid in designing interventions^19–21^. Pancreatic DTs, representing an “artificial pancreas”, can largely automate the decision algorithms for insulin administration, leading to control and reduction of long-term consequences of type I diabetes. The clinical success achieved with the artificial pancreas proves that the DT paradigm can profoundly change medical care and improve human health^22–25^.

Several factors have limited the development and adoption of in silico simulations of the immune system to improve patient care directly. We still need a complete understanding of the immune system’s health, disease, and therapy response functions. To progress, we must comprehensively leverage what we know and benefit from a wealth of data, tools, and algorithms to augment AI-enabled and mechanism-based simulations^26^.

**Fig. 1:**
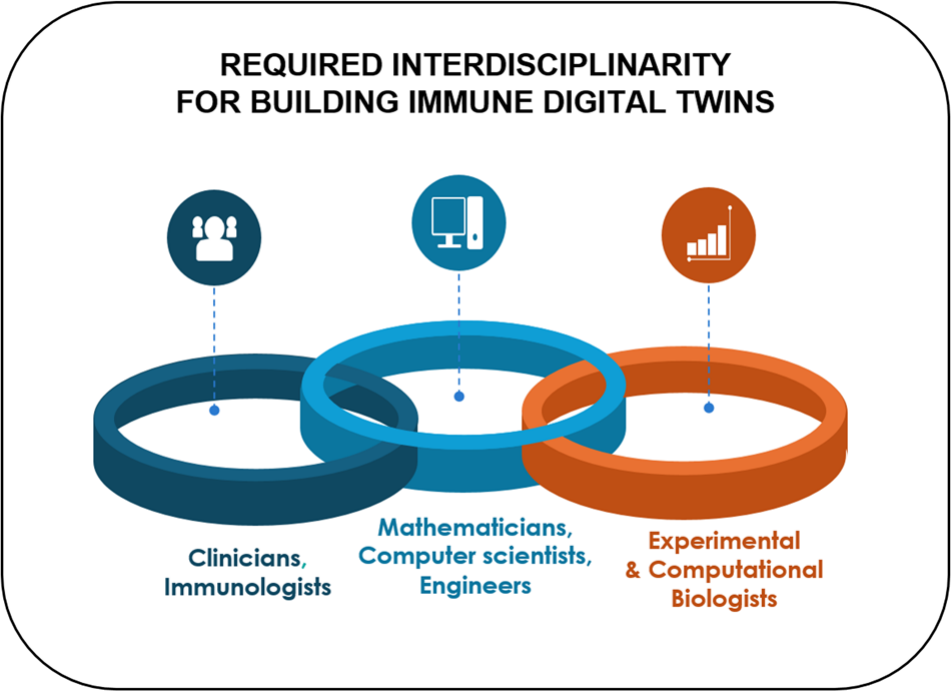
Interdisciplinarity as a key factor in Building Immune Digital Twins. Illustration of the collaboration among diverse stakeholders to establish an international and interdisciplinary community dedicated to the development and deployment of Immune Digital Twins. Modified template from https://youexec.com/.

Such a complex endeavour can only be achieved through a coordinated, combined effort of clinicians, immunologists, experimental and computational biologists, computer scientists, bioinformaticians, and mathematical modellers (Fig. 1). Initiatives to bring interdisciplinary and international consortia together are multiplying (Building Immune Digital Twins; Forum On Precision Immunology: Immune Digital Twins showcasing the need to bring together stakeholders from industry, pharma, biotech, start-ups, and bio-cluster sectors to form an active community on Building Digital Twins for the human immune system^27^. Bringing these communities together is one of the major challenges we face.

## Basic principles for designing IDTs

An IDT is a digital twin for a particular medical application with a significant immune system component. Besides specific characteristics for the “internal design and content”, the IDT should follow the FAIR principles^28–30^ to comply with best practices and community guidelines regarding large-scale and multi-scale models and be:

### Findable

The different IDT elements should be fully annotated and characterised by globally unique and persistent identifiers. They should be stored in appropriate data and model repositories, facilitating their retrieval. Their metadata should also be indexed in a searchable resource.

### Accessible

The different IDT elements and their metadata should be retrievable in an open and accessible manner following a standard communication protocol. The metadata should be available even if the IDT elements are inaccessible.

### Interoperable

Interoperability will allow the IDT to process information from multiple heterogeneous sources, ensuring a seamless flow of information. Standard input and output formats and the use of accessible programming languages and environments will help towards its adoption and usability. The IDT design requires a concerted effort by the systems biology community to adopt and implement suggested community standards, such as Systems Biology Markup Language (SBML)^31^ for mathematical model exchange, Systems Biology Graphical Notation (SBGN)^32^ for model visualisation, Biological Pathway Exchange (BioPaX)^33^ for pathway descriptions, and Simulation Experiment Description Markup Language (SED-ML)^34^ for simulation specifications. IDTs will likely use various modelling platforms, including tools that support ODE, agent-based, discrete, stochastic, or data-driven models. Not all of them are currently supported by community standards. Thus, there is a critical need to create standards for model specification for a much broader class of models through close collaboration and discussions with the COMBINE community^35^. A helpful resource is also the EDITH standards collection for Virtual Human Twins in Health^36^.

### Reusable

All IDT elements should be described in detail, comprising multiple attributes using standard metadata structures. Naming and annotating DT elements should be orchestrated by existing ontologies and newly designed controlled vocabularies, where needed. Transparency and accuracy in the description are necessary for maximising reusability. The aspects of scalability and modularity are particular to the IDT field of reusability. The IDT should be:

#### Modular

A modular IDT architecture allows for the integration of different components and models. Each IDT module can be derived from previously built models designed to represent specific aspects of the immune system; the modules can be designed from scratch to match unanswered questions, enabling flexibility to mix and match models as needed. This architecture supports easy updates, replacements, or additions. It allows the integration of new data and new data types and formats as they are discovered or developed, ensuring that the IDT can adapt to new research findings and evolving medical and biological knowledge, keeping it always up-to-date^37^. Ideally, the IDT design should be based on standardised, well-annotated modules that can be assembled into adaptable models. Note that the modelling community has long recognised^38^ that constructing a model in a plug-and-play fashion is a natural approach to managing model complexity and offers additional opportunities, such as the potential to reuse model components. In particular, the SBML Model Composition package (SBMLcomp)^39^ was developed to enable a modeller to include submodels within an enclosing model and edit, delete, or replace elements of that submodel. The concept of modularity in IDTs is similar to using containers and container libraries in bioinformatics frameworks^40^.

#### Scalable

Scalability is indispensable for integrating the different computational modules accounting for different scales and the computational power demanded for the simulations. A successful IDT requires a clear multilevel and multiscale organisation of the immune response, allowing for simpler surrogate models when complexity is unnecessary^41,42^ or computing resources are scarce. Additionally, the IDT infrastructure should be able to respond to an increase in data, number of models, or size of models relative to the immune system and the pathological context under consideration. It should include connections to HPC and cloud computing^43^. While supercomputers represent hardware-enhanced machines, HPC uses distributed resources to combine storage, applications, computational power, and network resources. Cloud computing refers to delivering services over the internet to facilitate access to resources and scaling. The combination of HPC and cloud computing could accelerate simulations at a large scale, thus significantly reducing the time to market for an IDT prototype. Furthermore, scalability, modularity, and interoperability should allow researchers to use IDTs at different complexity levels and with different computation resource needs. Alternatively, surrogate models of highly complex IDTs^41,42^, methods like model order reduction^44^, or the use of precomputed scenarios might be helpful to simulate IDTs in resource-limited settings. In addition, as a best practice in this context, model developers are encouraged to conduct reproducible comparative tests at different levels of scalability. This approach accurately establishes the relationship between the accuracy and quality of the results and the computational resources used in each test. By providing comprehensive metrics, model developers enable end users to determine the minimum computational resources required for their specific use cases while achieving results comparable to those initially reported.

**Fig. 2:**
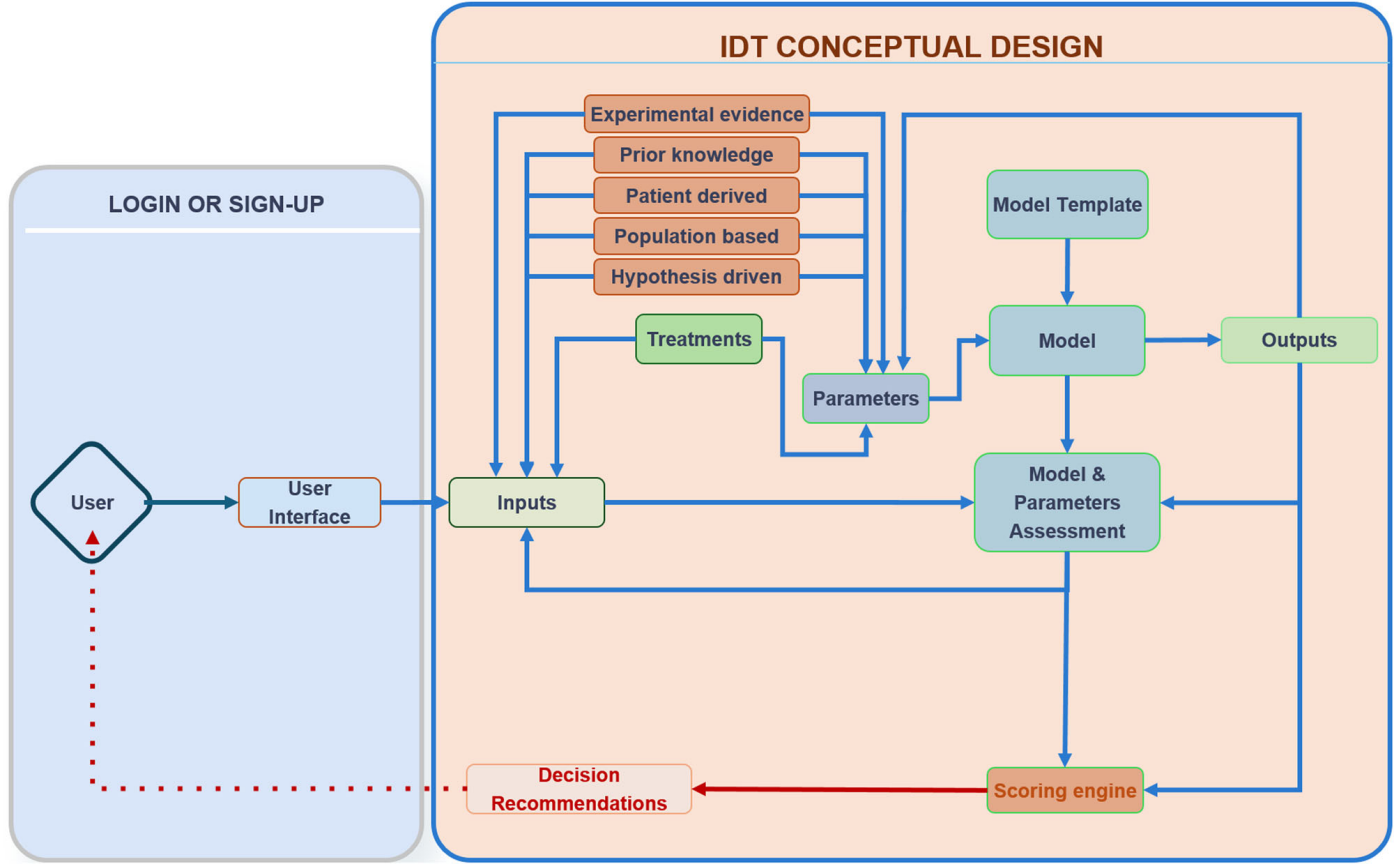
A minimalistic conceptual design of an IDT implementation. Producing a DT requires calibrating a computational model to data derived from a real-world patient. The connection to the real world is seen in the grey box to the left, where inputs of different types are generated for a particular individual and then passed to the virtual/computational model to personalise (“twin”) that general model to the specific patient. The “twinning” process involves parameterisation and a matching score to the real-world system by making predictions of how the real-world system propagates through time. This process iterates as new data becomes available and the DT is updated.

While the IDT should in principle be a two-way information system, ethical questions arise regarding the accessibility of the IDT predictions for patients^9^. In the proposed schema, the decision is not directly accessible by the patient, and it implies the presence of a control point, where a clinician (expert) uses the IDTs in silico results to make an informed decision that is then communicated to the patient. The level of accessibility to the IDT’s predictions should be controlled, and this can be addressed with different user categories having various types of rights. In Fig. 2, we offer a conceptual design of an IDT implementation.

## Integrating Artificial Intelligence (AI) models with mechanistic models for IDT construction

Data-driven solutions can bring valuable insights when the precise mechanism of interest is unknown but sufficient data is available. Deep learning (DL) models are playing increasingly pivotal roles in various domains^45,46^. However, their application still presents challenges, such as (i) the need for large amounts of data and computing resources (ii) ethical and privacy issues, particularly concerning the potential misuse of AI models and the risk of perpetuating existing biases in patient data; (iii) a complex regulatory landscape across countries and institutions, which severely limits the sharing of sensitive human data; and (iv) the need for interpretability, especially in the context of the GDPR EU law, which highlights the *right to explanation* and the necessity of transparency and accountability in automated decision-making processes. Due to the latter, many algorithms are trained on small and homogeneous cohorts, leading to data overfitting and limited generalisability to new patient groups. Innovative ML methodologies are emerging to combat these challenges. For instance, generating synthetic data informed by mechanistic knowledge offers a way to augment datasets and mitigate data scarcity and imbalance^47^.

Furthermore, models can be contextualised to represent a wide range of demographics and conditions, enhancing the diversity in patient population representation^48^. Foundational predictive AI models, built upon extensive multi-modal data encompassing scientific texts, molecular datasets, and biomedical knowledge graphs, are emerging in biology and show great potential in facilitating all aspects of model engineering, from biocuration to training model parameters^49^. Transfer learning, which involves pretraining models on larger datasets before fine-tuning with specific data, has been successfully implemented in data-scarce applications^50^. Federated learning, a collaborative learning approach that tackles data sharing and privacy issues by keeping data localised while enabling collective model training^51,52^. Lastly, the field of Explainable AI (XAI) is advancing solutions to identify data and algorithmic biases and to derive new insights from black-box models^53^, facilitating more reliable, safer and interpretable predictions of the immune response upon perturbations or treatments. Finally, the current approach to omics data analysis, especially in single-cell data science, is undergoing a significant transformation with the new generation of generative^54^ and causal AI methods^55^. These methods effectively bridge the gap between the data-driven approach^56,57^ and the mechanistic modelling, marking a notable shift in the traditional distinction between them.

A promising avenue for future development is the integration of AI and multiscale mechanistic models^55^. Mechanistic models excel in inferring causal relationships based on known biological mechanisms^58,59^, while AI models can help identify patterns and correlations within extensive datasets^20^. Hybrid IDTs could combine the robustness and interpretability of mechanistic models with the capability of AI models for extracting information from large data sets. Furthermore, hybrid models can address data scarcity while enhancing the robustness of the model, as exemplified in physics with physics-informed neural networks (PINNs), which showed that constraining neural networks with prior knowledge improves accuracy and generalisability even in data-limited scenarios^60^. However, biological systems are typically described in qualitative terms, and how to effectively integrate qualitative prior knowledge with quantitative and mathematical models requires further investigation. Despite the difficulty of integrating qualitative knowledge into deep learning models, proof of concept cases have already been published, such as pathway-aware multi-layered hierarchical networks for cancer patient classification^61^ or visible neural networks that can reproduce the inner workings of eukaryotic cells^62^. While further research is essential, hybrid IDTs could be particularly effective in predicting and suggesting therapeutic interventions targeting specific mechanisms^63^.

**Fig. 3:**
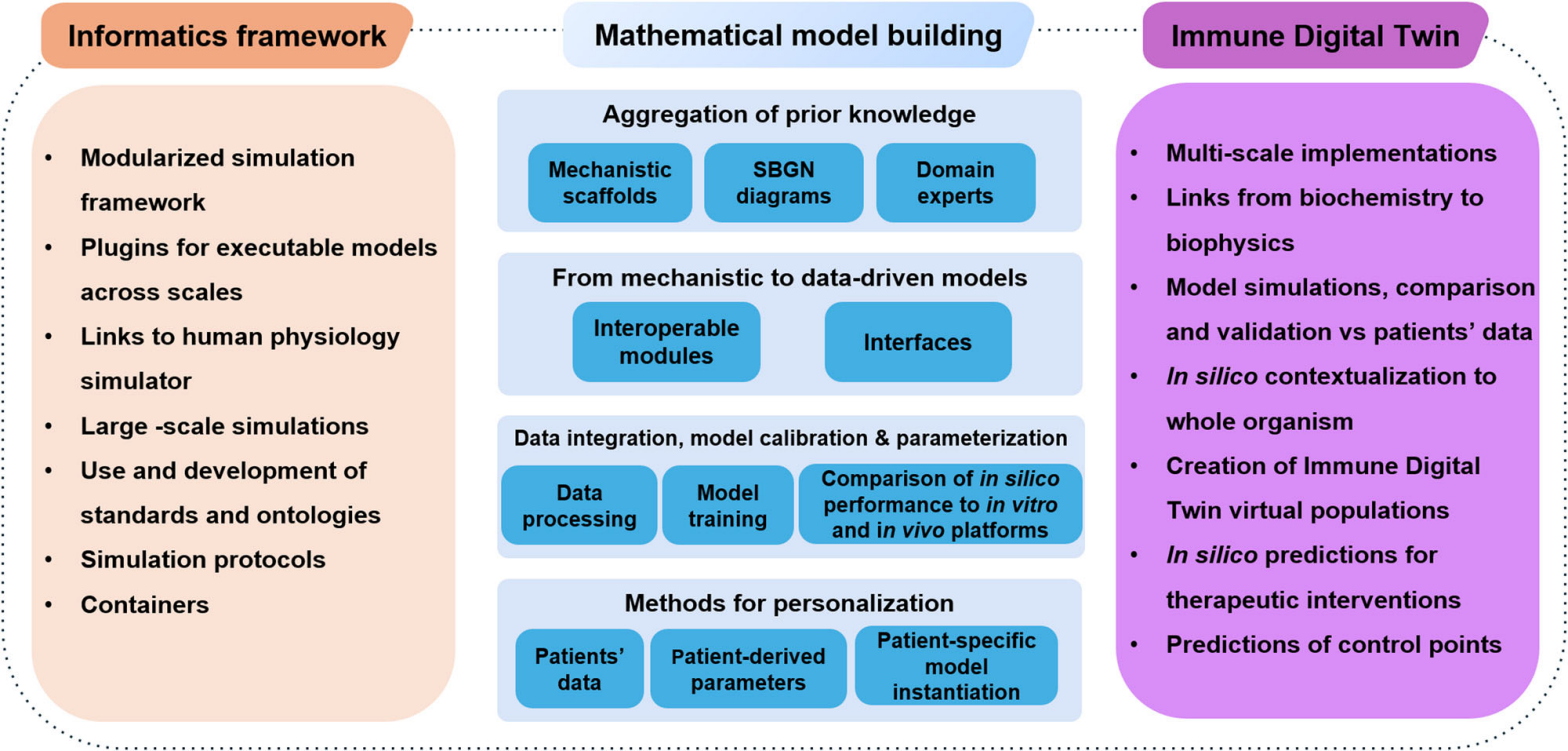
Different steps across scientific fields for a full-circle IDT implementation. Stepwise and domain specific steps for the implementation of IDTs. The building blocks of each scientific domain can be developed independently. However, attention to interoperability, use of common standards, and compliance with the FAIR principles will accelerate the building of IDTs that cover most of the technical/ methodological needs.

To summarise, efficiently building Digital Twins of the human immune system requires advancements in informatics and mathematics to accommodate the emergent needs that follow such an endeavour (Fig. 3). To bring IDTs to reality, one would need to leverage the progress in computational biology, AI, and the development of sophisticated integrative methods for low and high-throughput biological data spanning many biological layers to produce a robust ecosystem where data analysis and modelling could be directly linked to patients’ data, at both clinical and biological levels. Several building blocks can be developed independently, ensuring that standards, FAIR principles, and interoperability are factored in as we move towards the bigger picture.

## Implementing Immune Digital Twins in the study of complex human pathologies

**Fig. 4:**
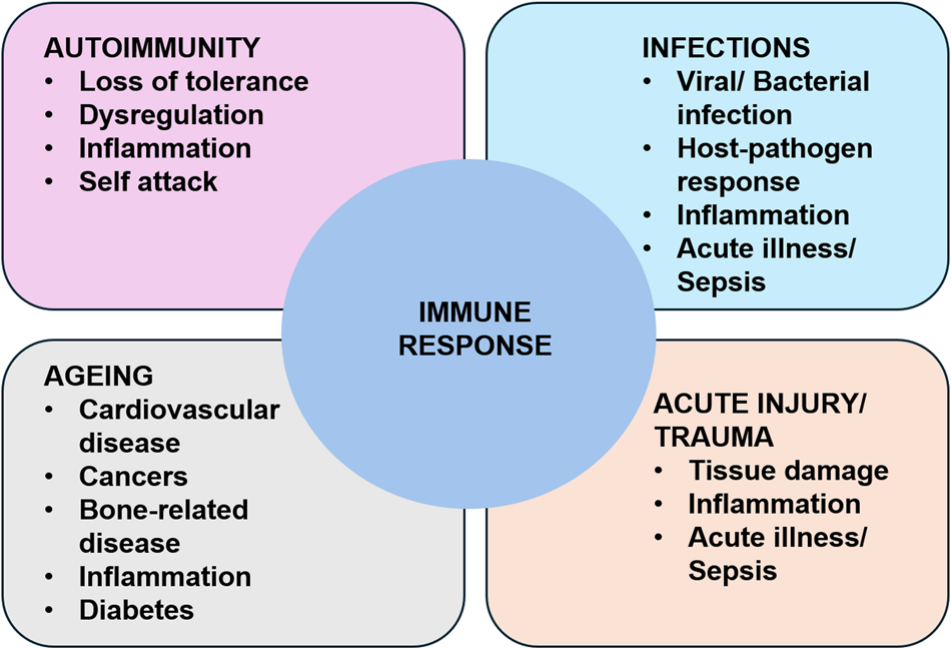
Human immune response in various pathologies. Immune/Inflammatory functions and their relationship to various classes of diseases: Autoimmune diseases in which failure in non-self-recognition or negative feedback control of proinflammation leads to persistent inflammation and long-term tissue damage; Infections in which the immune response is responding to various types of microbes (viruses, bacteria and fungi); Ageing, where changes in the function of the immune response can lead to a host of diseases; Acute Illness, where a host of perturbations rapidly activates the immune response. Immune pathophysiological processes range in time scale from hours for acute illness and sepsis to years and decades in autoimmune diseases and cancer. We propose that nearly every disease process and its potential resolution involves to some degree, inflammation and immunity.

As a causal and modulating factor, the human immune system is central to several disease classes, including infectious, autoimmune, and cancer (See Fig. 4).

**Fig. 5:**
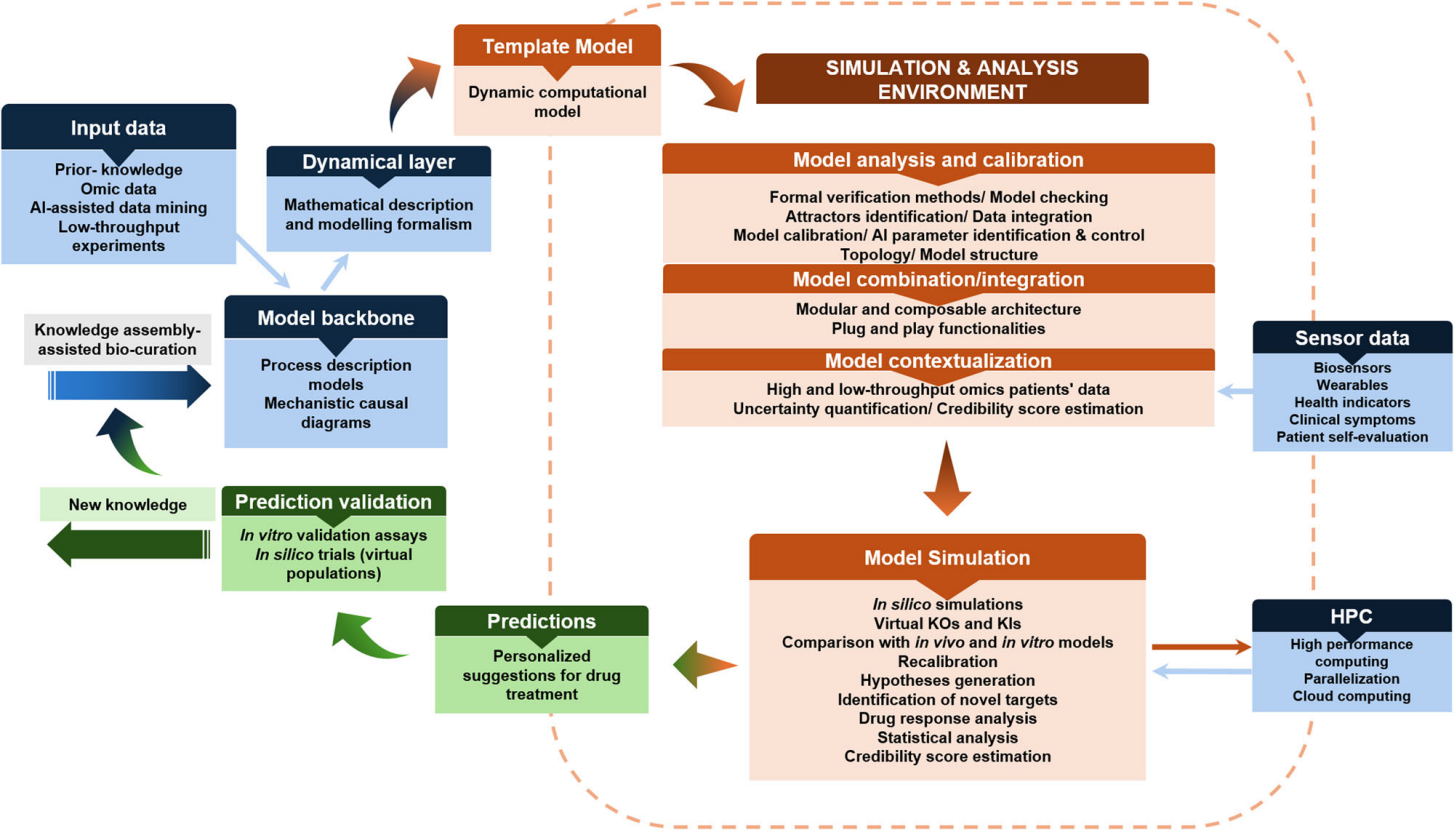
A bioinformatics ecosystem for data analysis, integration and modelling in IDT implementations. Stepwise process to obtain a DT implementation that accommodates different types of input data, integrative methods, and modelling formalisms to template model building, simulation, and analysis to create personalised instantiations for therapeutic interventions. The predictions can be tested using in vitro assays using humanised cellular systems (like organoids) and in silico population trials.

A defining feature of the immune system is that it operates across scales, bridging molecules to organs’ dynamics and spanning timescales from seconds to weeks, months, or years. This implies that digital twins incorporating immune system functions must be multiscale by default. The ubiquity of the immune system across a host of pathophysiological processes and the multiscale nature of how that pathophysiology manifests point to two classes of challenges when developing the infrastructure for implementing IDTs. The first is related to representing the relevant biology to be incorporated in a specific “fit-for-use” IDT while taking advantage of a shared knowledge base regarding the various components of the immune system. While having a comprehensive computational representation of the immune system that can directly model across different disease processes is an aspirational goal, in initial implementations of IDTs there will be specific choices made regarding what parts of the immune system will be incorporated into a specific IDT; the examples below will provide some insight into how this might be handled. The second challenge is technical: how should such IDTs be implemented? While multiscale modelling technology in biomedicine has made significant progress over the last decade^64^, many problems need to be solved, from software engineering to mathematics, including methodological challenges in sensitivity analysis or uncertainty quantification^65^. Specific use cases will direct the degree of representation and detail required for the IDT. A significant endeavour is identifying the mechanisms of interest and the core application (“fit-for-use”). The basic steps required for a full-circle IDT implementation are shown briefly in Fig. 5, and the examples of four fit-for-use IDTs are discussed afterward. In short, regardless of the pathology at stake, building a Digital Twin needs to address at least two things: pathology-specific events and the immune response to these events. From a more practical point of view, there are several distinct steps that one could follow to reach a mechanistic DT implementation, with adjustments and adaptations where possible to fit the purpose.

The following section presents four paradigms of potential DT implementations for four characteristic pathologies, including intra- and inter-cellular interactions expanding to organ levels and their communication with the immune system. All paradigms are based on existing efforts, published models, and/or disease approaches for DT, extended to account for multiple scales and frameworks. We call them paradigms to stress the goals and approaches, not the specific implementation. These paradigms include infectious pneumonia, rheumatoid arthritis, sepsis, and cancer.

### *Example 1:* Infectious pneumonia Immune Digital Twin (IP-IDT) paradigm

#### Background

Pneumonia is among the most common human diseases and the 4th leading cause of death. Infectious pneumonia inflames the air sacs in one or both lungs, which can quickly become life-threatening. Pathogenic insults such as viruses, fungi, or bacteria can cause the lungs’ air sacs (alveoli) to become inflamed and filled up with fluid or pus. A robust immune response is crucial for the clearance of the pathogen and the resolution of inflammation. However, an overpowering response can lead to acute respiratory distress syndrome (ARDS). Alveolar macrophages are the sentinels of the alveoli; their functions are broad, e.g., phagocytosis, clearing debris, resolution of inflammatory responses, and tissue remodelling^66^. Pulmonary macrophages are diverse, including tissue-resident alveolar macrophages that maintain immune balance and monocyte-derived alveolar macrophages that adapt to the microenvironment^67^. Recently, alveolar epithelial cells were found to actively participate in innate immunity by directly communicating with alveolar macrophages, phagocytosis of pathogens, and/or recruiting other leucocytes to the injury site. If the pneumonia lasts several days, adaptive immunity, such as T-cells and B cells, must be considered^68^.

#### Objective

Current ICU risk calculators accurately predict the likelihood of death but do not provide actionable information about therapeutic interventions in a particular patient. This paradigm aims to develop a computational model that encodes disease-relevant *biological mechanisms* and is *dynamically recalibrated* as new patient data becomes available in the clinic. The pneumonia DT can be used at any given time to simulate different interventions and help the critical care doctor optimise personalised interventions, such as timing and duration of antibiotic treatment based on patient status.

#### Implementation suggestion

The computational model underlying the IP-IDT needs to include mechanisms from the intracellular to the organism scale, as well as features of the pathogen. The epithelial cells lining the alveoli are involved in signalling events and coordinate the early immune response. They also sustain major damage as the infection develops. Immune cells, such as macrophages, dendritic cells, and NK cells, are recruited and secrete defensive substances. Fungal pneumonia starts in a highly localised fashion in immunocompromised patients, and the infection progresses slowly compared to bacterial pneumonia. To simulate pharmaceutical interventions, the intracellular and tissue scales are particularly important. Other interventions, such as prone positioning of patients in ICU beds or mechanical ventilator assistance, require lung physiology representation. A suitable implementation of an IP-IDT could consist of Ordinary Differential Equations (ODE) or discrete models at the intracellular scale, agent-based models (ABM) at the tissue scale and ODE models at the whole-organ scale.

#### Input data

In an ICU setting, routinely collected data include immune cell counts, cytokine levels, and regular blood draws. One can periodically obtain data from intubated patients through bronchoalveolar lavage characterising the lung environment, such as cell counts, infection severity, and epithelial damage. X-ray images are collected regularly, as well as occasional CT scans. Intracellular data are not likely to be collected, requiring the construction of surrogate models.

#### Potential impact

When fully developed, a pneumonia DT could provide a critical care doctor with a decision-support tool that provides actionable, personalised recommendations for a given patient at a given time.

### *Example 2:* The rheumatoid arthritis Immune Digital Twin paradigm (RA-IDT)

#### Background

Rheumatoid arthritis is an autoimmune complex disease affecting the human body’s articular joints. The disease is multifactorial, with genetic and environmental factors pivotal in the disease pathogenesis. RA’s aetiology is unknown, and the treatment is primarily symptomatic. The disease affects the immune system, mistakenly attacking the synovial lining of the joints, causing inflammation, cartilage destruction, and bone erosion. The autoimmune component is central; however, other mechanisms, both immunologic and tissue-derived, clearly contribute to its onset and progression^69^. If left untreated, the fulminant stage of the disease is described by a hyperplastic inflamed synovium, cartilage damage, bone erosion, and other systemic consequences^70^. The response rate to current therapies is around 40%^71^, demonstrating the pressing need for accelerating innovative and powerful technologies for personalised care.

#### Objective

Many cells and their cytokines play critical roles in the development of RA. The synovial compartment is infiltrated by leucocytes and the synovial fluid is inundated with pro-inflammatory mediators that are produced to induce an inflammatory cascade, which is characterised by interactions of fibroblast-like synoviocytes with the cells of the innate immune system, including monocytes, macrophages, mast cells, dendritic cells, and so on, as well as cells of adaptive immune system such as T cells and B cells. Endothelial cells contribute to the extensive angiogenesis. The fulminant stage contains hyperplastic synovium, cartilage damage, bone erosion, and systemic consequence. The destruction of the subchondral bone can eventually result in the degeneration of the articular cartilage as the result of a decrease in osteoblasts and an increase in osteoclasts and synoviocytes^70^. The main aim of the RA-IDT would be to digitally represent the interplay between resident cells of the joint and immune cells in RA, which leads to bone erosion, cartilage breakdown, and inflammation, to the level where the RA-IDTs can propose personalised therapy interventions. One major unmet need in the field is the understanding of response to therapy, especially for the non-responders to traditional medication.

#### Implementation suggestion

Hybrid modelling methods that allow for combinations of large-scale inter-cellular models with cell-level agent-based models could create a virtual joint. Recently, disease and cell-specific pathway models^72,73^ and dynamic models on the intra and inter-cellular level have been developed^74–79^ that could serve as the core components of an RA-IDT. Moreover, given some shared characteristics, especially regarding bone erosion and cartilage destruction, between RA and Osteoarthritis (OA), OA models could also be contextualised and implemented in the RA-IDT^80,81^. Modelling methods that couple signalling, gene regulation, and metabolic fluxes are now available^74,82^ and can be combined with omics data technologies to create personalised instantiations. For the RA-IDT, a combination of large-scale Boolean models that govern cellular behaviour, with agent based models (ABM) for accounting for the interactions of multiple cellular types, could be a suitable implementation. In ABM, agents may receive signals and input from the environment and their neighbouring agents, provide output to the environment and their neighbours, and make ‘decisions’ based on the input from around them and their internal, sub-cellular decision making rules. An agent may grow, proliferate, enter a quiescent state, express cytokines/chemokines or undergo apoptosis or necrosis in response to surrounding environmental conditions. A first attempt to link Boolean models with ABMs was done during the COVID-19 Disease Map initiative^83^.

#### Input data

Biosensors that can measure matrix degradation and bone erosion along with patient-reported outcomes and scores, could be used to assess the patient’s joint and bone health and monitor changes over time^84^. The RA-IDT could also be integrated with other technologies, such as imaging techniques or wearable non-invasive sensors (smart watches, smartphone applications), to provide a more comprehensive picture of the patient’s joint and overall health.

#### Potential impact

Complex diseases are associated with a heavy societal burden that stems from patients’ disabilities and health conditions and the economic costs which come with it. The RA-IDT could help in identifying novel therapeutic combinations tailored to the needs of individual patients, especially beneficial for the ones not responding to traditionally administered medication. In addition, it could provide valuable insights into the disease pathogenesis and help identify new drug targets.

### *Example 3*: The sepsis-IDT paradigm

#### Background

Sepsis is a syndrome in which a disordered immune response to severe infection or injury leads to early proinflammatory collateral tissue damage/organ dysfunction^85^ and later immune incompetence, leading to increased susceptibility to nosocomial infections^86^. As sepsis is a systemic disease, a sepsis-IDT will necessarily include the organs at risk: the immune system, lung, liver, kidney, gut, and cardiovascular system.

#### Objective

The primary goal of the Sepsis-IDT is to provide the capability to treat sepsis by multimodal adaptive modulation of a patient’s underlying cytokine milieu (“fit-for-purpose” as per the NASEM report). It will account for sepsis’s heterogeneity and dynamic complexity by having an ongoing data link between the virtual and real twin and informing control/guiding therapy^1^.

#### Implementation suggestion

The clinical time scale of sepsis (hours to weeks) focuses on the acute aspects of the innate immune response and its initial transition to adaptive immunity. An example of an existing computational model for this can be seen in ref. ^87^. Furthermore, sepsis manifests in dysfunction of multiple organs, and thus, each organ system can be cast as a module of the system-level Sepsis-IDT. An early example of this approach simulates the gut-lung axis in sepsis. It consists of modular agent-based models of tissue/organ-specific epithelial cells interacting with and connected to an agent-based model of endothelial cells and circulating immune cells.^88^. These existing examples of dynamic multiscale molecule-to-organ integration form the basis of an initial Critical Illness Digital Twin (CIDT)^89,90^. This CIDT would be used to train (off-line) an artificial intelligence controller (AIC) offline, as described in refs. ^90,91^. This digital twin-trained AIC would be the “brain” of an integrated cyber-physical system that monitors real-time plasma cytokine/mediator levels (using existing technologies as described in ref. ^92^ and uses the AIC to guide the administration of different amounts of pro- and anti-inflammatory mediators/monoclonal antibodies to steer an individual patient back to a state of health.

#### Input data

The ongoing data link between the virtual and real world, per the NASEM DT definition, can be accomplished for the Sepsis-IDT via a suite of clinical and laboratory measurements providing dynamic molecular profiling of the patient’s immune state as reflected by circulating inflammatory mediators^92^ and determinants of trajectories of organ function. For the latter, the Sequential Organ Failure Score (SOFA)^93^ and its variants^94^ can be generated by readily obtainable clinical measurements and sequentially measured to update the IDT. The current version of the CIDT is poised for testing in a sufficiently complex animal model of sepsis, but should the performance of this cyber-physical system be insufficient, the development loop can both refine the Sepsis-IDT and guide sensor/assay developments as per guidance from the NASEM report.

#### Potential impact

Given that the current inability to effectively modulate the inflammatory/immune dynamics in sepsis is due in significant part to the heterogeneity and dynamic complexity of the disease process^95^, the personalised and adaptive control capabilities offered by the digital twin paradigm point to the potential therapeutic benefits of a successfully implemented Sepsis-IDT.

### *Example 4:* The onco-IDT paradigm

#### Background

The ability to evade immune surveillance and destruction is a hallmark of cancer^96^. Immunotherapy approaches have significantly improved cancer outcomes for several cancer types^97^. During oncogenesis, the immune system activates a multifaceted response involving innate and adaptive immune systems. However, cancer cells and their environment progressively evade immune surveillance by down-regulating major histocompatibility complexes (MHC) and upregulating immune checkpoint proteins^98,99^. Cancerous, immune, and stromal cells are the critical components of the tumour microenvironment (TME), and their interplay represents a complex system. The US National Cancer Institute and the US Department of Energy have started to explore the development and implementation of predictive Cancer Patient Digital Twins for personalised treatment^100^.

#### Objective

The main aim of Onco-IDTs would be to provide a computational representation of the patient’s cancer disease, including the interactions between cancer cells, tumour-associated cells, and the immune system. An ideal Onco-IDT should have the ability to be calibrated to patient’s data and used for clinical decision support. An Onco-IDT should include elements like the TME, neo-angiogenesis, pre-metastatic and metastatic conditions, and system-level information like blood and lymphatic transport. Today, personalised therapy in oncology is making progress in identifying cancer-driver mutations for each patient^101^ or cellular patterns linked with disease state/progression^102–104^. Successful implementations of in silico mechanistic models led to the identification of optimal treatments with minimal toxicity in melanoma and breast cancer^105,106^. Recently, patient-specific Boolean models of signalling networks were used to guide personalised treatments^107^.

#### Implementation suggestion

A possible implementation of an Onco-IDT can draw inspiration from the multiscale model of the different modes of cancer cell invasion described in ref. ^108^. The framework includes agent-based modelling and continuous time Markov processes applied on Boolean network models. The model is focused on cell migration considering not only spatial information obtained from the agent-based simulation but also intracellular regulation obtained from the Boolean model. It could be expanded to account for more complex phenotypes and interactions of tumour cells with cells from TME and immune cells, and also adapted to include clinical features and patient-derived characteristics. An Onco-IDT could be employed to optimise treatments and treatment combinations and potentially predict response to a particular treatment for individual patients.

#### Input data

Extensive cell phenotyping, genetic testing, and sequencing of tumour material is possible but requires tissue obtained by biopsy. The biopsy-based molecular subtyping provides direct data on a tumour’s current state and microenvironment, and single-cell techniques are powerful tools to capture natural and pharmacologically induced tumour immunity^109^. However, cancer genomics-guided approaches harnessing or targeting the immune system are still in their infancy, and surrogate markers are used. Also, biopsies are invasive and usually reserved for diagnostics, limiting their ability to track tumour development over time and sample intra- and inter-tumoral heterogeneity. Data and measurements from non or semi-invasive interventions, such as electronic health records, radiological imaging, and serological (circulating tumour cells and circulating tumour DNA) or molecular data that could inform about the patient’s inflammatory state, can be integrated with the biopsy data points. In addition, blood samples can provide insights into relevant drug pharmacokinetic (PK) processes, while wearables can offer additional information regarding vital signs, body temperature, and physical activity levels.

#### Potential impact

An effective Onco-IDT will provide oncologists with a dynamic clinical decision-support platform, aiding prognosis and disease management. More specifically, the Onco-IDT could contribute significantly to prognostic predictions regarding disease course, considering factors like metastatic capacity and patient survival. Additionally, it could offer actionable insights concerning therapeutic interventions, involving selecting the most effective therapy that maximises benefits while minimising side effects and adverse outcomes. Therapeutic decisions might also be optimised to select effective monotherapies versus combination therapies, drug doses, and treatment schedules.

These paradigms outline four scenarios where digital twins could be deployed for treatment optimisation. The sepsis and pneumonia paradigms take place on very short time scales, days or weeks, whereas the oncology and autoimmune paradigms focus on time scales of months or years. Yet, the implementation of IDTs represents a key enabling technology for the personalisation and optimisation of treatment. While the pathological characteristics are very different, the technical modelling challenges are similar. Besides data challenges, from obtaining appropriate data to interpreting them and turning them into model parameterisations, the modelling scaffolds and implementations could follow shared reasoning. For example, for molecular level simulations: one could use networks, Boolean models, ODE-based models, discrete logic-based models; for cellular level: networks, Boolean models, ODE-based models, discrete logic-based models, multiscale models, hybrid models; for cell-cell communication and tissular level: Agent-based models, multicellular models, Boolean models, ODE-based models, discrete logic-based models; for organ level: structural, biophysical, biomechanical models, ODE models, and for system level: multiscale models, biophysical models, hybrid models, human physiology engines. In Table 1 we list available software and platforms for different modelling types and scales.

**Table 1:** Computational modelling software and simulation platforms that can be employed to build models of different scales

Computational modelling		
Scale	Tool	Type
**Single scale (Molecular/Cellular)**	Aeon	Boolean
	Bio Model Analyzer	Logic-based, multivalued
	Biocham	Boolean
	BioNetGen	Rule-based/ODE/ABM
	BooleSim	Boolean
	BoolNetR	Boolean
	Cell Collective	Boolean
	CellDesigner	ODE
	CellNetAnalyzer	Boolean
	CellNOpt	Boolean
	COMSOL Multiphysics	PDE
	COPASI	ODE/SDE/Gillespie/steady-state solvers
	GINsim	Boolean - genetic regulatory networks
	JWS online	ODE
	Kappa	Rule-based/ABM
	MaBoSS	Boolean, stochastic
	MATLAB toolkit	ODE
	M-cell	ABM cellular spatial
	NERDSS	ABM spatial
	PhysiCell	ABM cellular spatial
	PyBoolNet	Boolean
	Smoldyn	ABM spatial
	SpringSaLaD	ABM spatial
	Tellurium	ODE
	Virtual Cell (VCell)	ODE/PDE/SSA/ABM
	WebMaBoSS	Boolean models
**Metabolic**	CellNetAnalyzer	Flux Balance Analysis
	COBREXA	Flux Balance and Flux Variability Analysis
	Escher	Flux Balance Analysis
	KBase	Flux Balance Analysis
	Metabolizer	Flux Balance Analysis
	MetaLo	Metabolic analysis of logical models extracted from maps
	RAVEN	Genome scale metabolic modelling
**Multiscale**	Chaste	Molecular + Cellular, supports CellML
	CompuCell3D	Molecular + Cellular
	Morpheus	Molecular + Cellular
	NetLogo	Molecular + Cellular
	PhysiBoSS	Molecular + Cellular
	Tissue Forge	Molecular + Cellular
	Vivarium	Merging multiple scales
**System level**	BioGears	Human Physiology
	Physiome Project	Virtual Physiological Human
	Pulse	Human Physiology
	Universal Immune System Simulator	Generic Immune System simulator

Additional useful resources, such as pathway editors, databases, visualisation software, simulation environments and repositories, as well as ML/AI tools, that can be used in various steps of the IDT building can be found in Supplementary Data 1.

## IDTs in drug discovery

Drug development is costly and slow. The costs include early research and discovery costs through clinical development, regulatory approval, and post-marketing surveillance. Most candidate targets and drugs experience failure in the early stages, contributing significantly to the overall cost of delivering more successful candidates. Therefore, optimising these earlier stages holds transformative potential in the pharmaceutical industry. A strong consensus among experts supports the opinion that the involvement of digital twins in this transformation can be essential^110^.

The introduction of a specialised form of drug development digital twins (DDDT) has the potential to be a game-changer^111^. Moreover, we can envision further specialisation of DDDTs based on various tasks in the early drug discovery process. These tasks include 1) identifying targets and their combinations, as well as determining the most promising treatment modalities (encompassing not only small chemical compounds but also antibody-drug conjugates, various types of biologics, and gene or cell therapies); deciding on the level at which the target should be affected (whether directly, through its RNA, or its involvement in protein-protein interactions), 2) experimental target and drug validation, aiding in identifying the most informative experimental systems (such as cell lines or organoids) and experiment designs, 3) repurposing drugs for alternative indications in case of a failure for the primary one, 4) delivering drugs by integrating pharmacokinetic models into the global in silico models of treatment and taking into account safety aspects early in the process, 5) finally, there might be flavours of DDDTs aimed at optimising the process of drug production, with notable examples like Sanofi exploring the use of digital twins for vaccine manufacturing^112^.

All these DT specialisations require specific designs, functionalities, and connections to the existing wealth of public and proprietary data. Furthermore, virtual populations of patient DTs can be used to run in silico clinical trials that can accompany or be used to design real-life trials^113^. One recent example is the Universal Immune System Simulator (UISS)^114^. The European Medicines Agency (EMA) provided a letter of support for the use of the UISS as a simulation platform to predict how the circulating interferon-gamma (IFNγ) changes over time as a function of the treatment dose in a cohort of virtual patients to select the doses to be tested in escalating dose phase IIa trials of new therapeutic whole cell / fragmented based vaccines against several diseases^115^. Whereas more work is required before qualification advice can be given, it does show that EMA believes this is a genuine possibility. Recently, a book was published focusing on best practices for using computational modelling and simulation in the regulatory process of biomedical products, showcasing the need to address policy and implementation early on in the DT design^116^.

## Regulatory and ethical aspects of IDTs

Regulatory stakeholders like the Food and Drug Administration (FDA), European Medicines Agency (EMA), and other national agencies have expressed their acceptance of simulation-derived results, as evidenced in the submission dossiers. FDA started the Model-Informed Drug Development (MIDD) pilot program about a decade ago^117^. Owing to the success it found in advancing medicinal product development, the FDA has now established MIDD meeting formats for the fiscal years 2023-2027, welcoming modelling and simulation applications at various stages of product development, especially in the domain of rare/orphan and paediatric indications^118,119^. EMA has also evaluated the modelling and simulation approaches earlier through the Modeling and Working Party to increase awareness across European national authorities.

Moreover, National funding agencies in Europe and the US encourage the use of innovative digital technologies as alternative methods for animal experiments.

If IDTs are to form an intrinsic part of the medical decision processes, they must be classified as software devices for medical use. Accordingly, they must undergo a software development process that complies with the IEC 62304 standard, among others^120^. The FDA has also published guidelines regarding the credibility assessment of computational modelling and simulations in medical device submissions^121^. For a smooth transition from research-use-only software to medical software devices, meeting these requirements as early as possible in the development process and at a reasonable cost will be essential. Following established guidelines from the systems biology community^122–125^ for model specification, documentation, data file formats, etc., is the critical first step in ensuring compliance with regulatory requirements.

Digital twin technology and the AI era also raise ethical questions regarding the protection of personal data, their use, and their legacy, as well as the democratisation of such technology for the benefit of humanity^126^. The success of these technologies in health care depends on addressing three main ethical challenges: the right and exhaustive training of AI models; healthcare data management and respecting the privacy of patients; and encouraging patient trust in clinicians who use AI-based tools^127^.

Questions regarding the accessibility of the template and personalised models, the use of clinical data to train AI algorithms that could be commercialised, employers’ and insurance companies’ access to digital twins of employees, and the fate of a digital twin after the passing of the actual patient are open to discussion and debate^128^.

## Limitations and challenges

Digital twin technology for healthcare is advancing at a fast pace. The numerous scientific articles published in the last two years have tried pinpointing the multiple challenges the scientific community faces to bring DTs into the preclinical and clinical settings^4–18^. One of the most common issues is the reconciliation of the different scales in biology. While some fields have been progressing fast, especially in the cardiology domain^20^, the twin is built on the organ level (i.e. modelling the heart), focusing primarily on biophysical, structural, and biomechanical characteristics derived from imaging sources, electrocardiogram (ECG) databases and, in some cases, clinical data, including data from biosensors and wearable technology^129,130^. However, pharmaceutical treatments operate on the molecular and cellular level, and the absence of causal molecular interaction networks underlying the biophysical processes creates a critical gap. While not all DTs should span all layers and scales, building the technology and creating reproducible, scalable, and interoperable frameworks to link these scales when needed is a necessary step for moving forward.

Similarly, digital twins on the cellular or tissue scale often fail to scale up and provide links to full-body manifestations and clinical measurements as they focus on a few molecular or cellular processes. Conclusions extrapolated to an organ or patient level are often made based on a limited number of biomarkers or phenotypes. In this case, these sophisticated computational models are complex to implement in the clinical or preclinical setting, as they operate on a different level regarding routine clinical measurements and patient assessment.

**Fig. 6:**
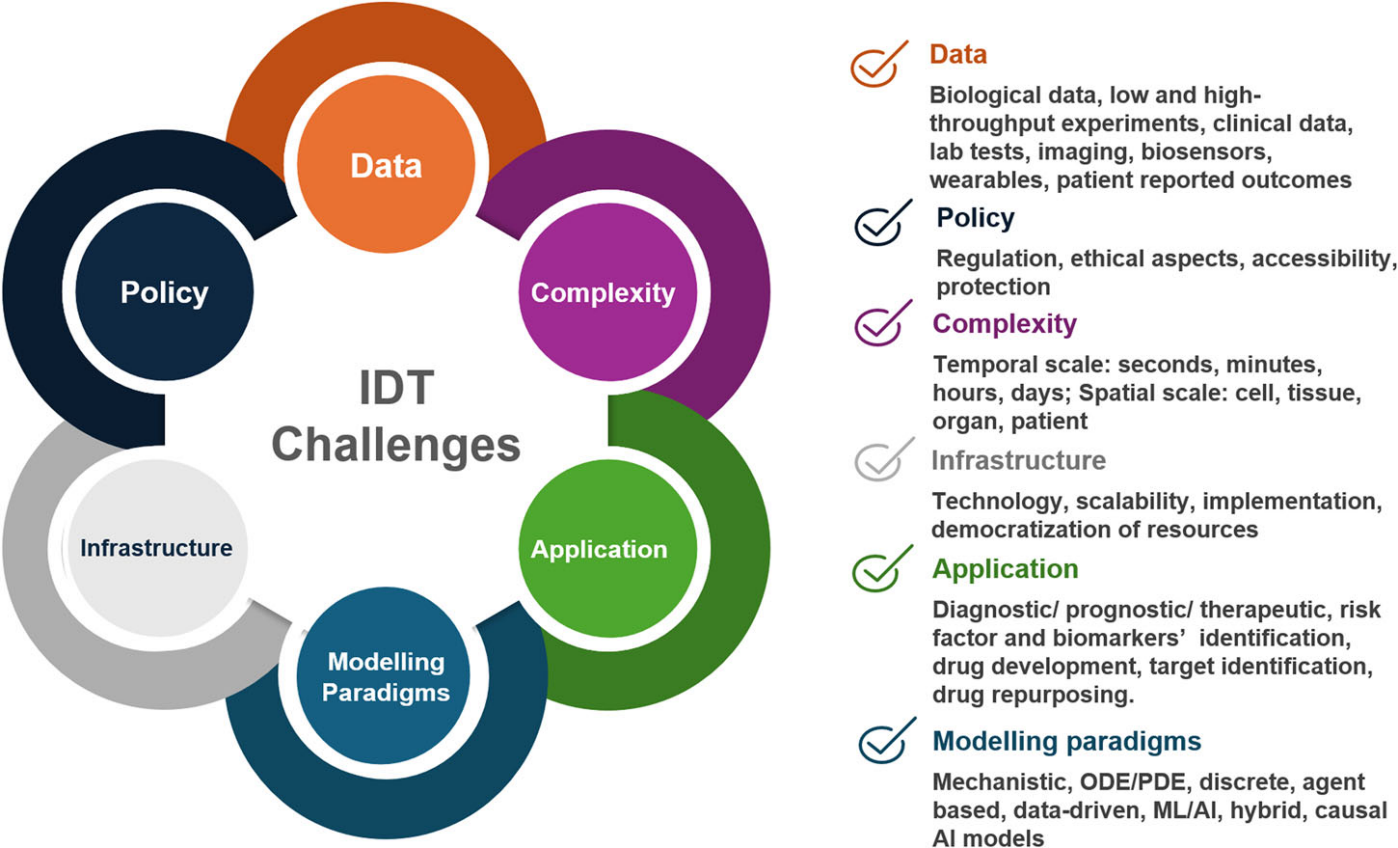
Key challenges in developing and implementing IDTs in pre-clinical and clinical settings. The implementation of IDTs require a community-driven approach to tackle challenges in data acquisition, analysis and integration, policy and data protection, methodological aspects to address complexity, dedicated infrastructure development. Tailor-made solutions are also needed to address specific unmet needs in different fields of application, and, lastly, robust and credible scalable modelling approaches for complex human pathologies that involve characteristic immune responses. Modified template from https://youexec.com/.

Building Digital Twins of the human immune system comes with some additional hardship. The immune system is inherently complex and operates on multiple scales, including organs and cells. Moreover, it is difficult to establish a “baseline” modus operandi that fits a general population. Focusing on specific pathological conditions, which have distinct localised and systemic manifestations of the dysregulation, and where the interplay between resident and immune cells is more straightforward to measure and quantify, might be the best approach for the first “proof of concept” IDT implementations. Moreover, as inflammation seems omnipresent in most disease settings, it could be seen as the core immune response that could be built and modelled in an adaptable way to fit most cases. Figure 6 summarises the challenges associated with DT development and implementation, especially those related to data, policy, complexity, infrastructure, application, and modelling paradigms.

## Perspectives

A change of mindset is needed to achieve tangible results in IDT technology. Traditionally, clinicians, immunologists, and experimental biologists identify hallmarks of the disease, disease biomarkers, and affected pathways, organs, and systemic manifestations. They also include in the study measurable factors used in the clinic and employ available experimental techniques to enrich the molecular, genomic, metabolic, and clinical profile of the patients. Bioinformaticians and computational biologists then analyse the data available and provide coherent links and possible abstractions that could capture the essential characteristics of the system. However, an early inclusion of the bioinformaticians and modellers in the study design could ensure that the minimal set of measurements for building a reliable model is factored in. Likewise, exchanges and discussions early on in a research project would allow for a maximum comprehension of the disease mechanisms and questions at stake. Besides IDT design and implementation, bioengineers can help identify and manufacture critical biosensor technologies that could be implemented into the IDT computational ecosystem. Partnerships with startups could accelerate the production of prototypes, and the industry could contribute by providing infrastructure for the necessary scaling and support for bench-to-market pilot studies.

Mechanism-based simulations, AI-enabled data integration, and subsequent experimental and clinical validation will allow for the iterative improvement of human immune system models. These models will become increasingly more accurate and robust in their capacity to simulate the human immune system’s reactivity against insults and dysregulation in disease and predict potential pharmacologic intervention points at different scales.

## Supplementary information

Supplementary Table 1
